# Deactivation of the Default Mode Network as a Marker of Impaired Consciousness: An fMRI Study

**DOI:** 10.1371/journal.pone.0026373

**Published:** 2011-10-19

**Authors:** Julia Sophia Crone, Gunther Ladurner, Yvonne Höller, Stefan Golaszewski, Eugen Trinka, Martin Kronbichler

**Affiliations:** 1 Neuroscience Institute and Centre for Neurocognitive Research, Christian-Doppler-Clinic, Paracelsus Private Medical University, Salzburg, Austria; 2 Department of Psychology and Centre for Neurocognitive Research, University of Salzburg, Salzburg, Austria; 3 Department of Neurology, Christian-Doppler-Clinic, Paracelsus Private Medical University, Salzburg, Austria; Institute of Automation, Chinese Academy of Sciences, China

## Abstract

Diagnosis of patients with a disorder of consciousness is very challenging. Previous studies investigating resting state networks demonstrate that 2 main features of the so-called default mode network (DMN), metabolism and functional connectivity, are impaired in patients with a disorder of consciousness. However, task-induced deactivation – a third main feature of the DMN – has not been explored in a group of patients. Deactivation of the DMN is supposed to reflect interruptions of introspective processes. Seventeen patients with unresponsive wakefulness syndrome (UWS, former vegetative state), 8 patients in minimally conscious state (MCS), and 25 healthy controls were investigated with functional magnetic resonance imaging during a passive sentence listening task. Results show that deactivation in medial regions is reduced in MCS and absent in UWS patients compared to healthy controls. Moreover, behavioral scores assessing the level of consciousness correlate with deactivation in patients. On single-subject level, all control subjects but only 2 patients in MCS and 6 with UWS exposed deactivation. Interestingly, all patients who deactivated during speech processing (except for one) showed activation in left frontal regions which are associated with conscious processing. Our results indicate that deactivation of the DMN can be associated with the level of consciousness by selecting those who are able to interrupt ongoing introspective processes. In consequence, deactivation of the DMN may function as a marker of consciousness.

## Introduction

Patients with a disorder of consciousness (DOC) like patients in the vegetative state and patients in minimally conscious state (MCS) have survived severe brain injury to a state of wakefulness with no or minimal awareness of themselves and their environment (see Laureys et al. [1] and Owen et al. [2] for further review). As postulated by the European Task Force on Disorders of Consciousness to avoid associations with a vegetable-like condition [3], we will further refer to patients in the vegetative state as patients with unresponsive wakefulness syndrome (UWS). Patients with UWS are, by definition, not conscious aware and show no evidence of voluntary behavior; whereas patients meeting the diagnosis criteria of MCS have recovered from UWS demonstrating inconsistent but observable signs of consciousness [4]. Accurate diagnosis is very challenging. Studies report that more than one-third of these patients are misdiagnosed [5], [6], [7]. In fact, 9% of patients with DOC were able to willfully modulate their brain activity during mental-imagery tasks in a functional magnetic resonance imaging (fMRI) study [8]. The high percentage of misdiagnoses encouraged many scientists to test different paradigms in imaging and electrophysiological studies to find reliable markers for diagnosis and prognosis [9], [10], [11], [12], [13], [14], [15], [16], [17], [18], [19], [20], [21]. In the course of these investigations, the so-called default mode network (DMN) has become an important focus of interest. Coherent spontaneous low-frequency fluctuations in the resting brain are organized into distinct brain networks such as the DMN [22] including medial parietal and frontal brain regions like the posterior cingulate cortex (PCC) and the ventral anterior cingulate cortex (vACC) [23]. In healthy subjects, a resting state network like the DMN is characterized by, first, high metabolism during resting state [24], [25]; second, functional connectivity during rest [26]; and third, deactivation during various attention-demanding cognitive tasks [27], [28]. Yet, only 2 of the 3 main features of the DMN have been investigated in a group of DOC patients. First, global cortical metabolism during the resting state is reduced by 40–50% in patients [16], and especially in medial parietal and frontal regions, metabolism is systematically impaired [29]. Second, Vanhaudenhuyse and colleagues [30] could demonstrate a reduced functional connectivity of the DMN and a correlation with the level of consciousness. The third feature of the DMN, task-induced deactivation, has only been assessed in a single-case study in which an UWS patient showed a reduced pattern of deactivation in regions of the DMN compared to the time of recovery 7 months later [31]. This finding implies that deactivation of the DMN may be related to conscious processing. Further, deactivation in medial parietal regions and medial frontal regions is hypothesized to reflect interruptions of introspective processes to engage in attention-demanding actions [32]. While resting-state connectivity is probably not directly related to different states of consciousness but to a more basic function of cognitive processing [33], deactivation of the DMN is a target-directed reaction to attention-demanding stimuli and, therefore, may clarify additional aspects concerning the state of consciousness. We assume that only those patients with more preserved cognitive functions will be able to interrupt ongoing mental processes and, therefore, show deactivation. Thus, deactivation of the DMN may provide additional information for diagnosis and may offer new insights on the functionality of the DMN throughout cognitive tasks and rest. In this study, the DMN has been investigated in 25 patients with DOC during a passive sentence listening task to explore the additional information value of group and single-subject task-induced deactivation patterns.

## Materials and Methods

### Subjects

The study was approved by the local Ethics Committee (Ethics Commission Salzburg/Ethikkommission Land Salzburg; number 415-E/952). Written informed consent was obtained according to the Declaration of Helsinki from all control subjects and from the families or guardianship of all patients. All participants were capable of the German language.

Twenty five age-matched healthy subjects (10 men and 15 women; mean age 49 years; age range 22–70 years) were recruited at the Paris Lodron University of Salzburg. None of the subjects had a neurological or psychiatric disease history. Thirty patients with DOC (10 patients in MCS; 20 patients with UWS) were examined for this study. Because of severe motion artifacts in both sessions, 5 patients (2 patients in MCS; 3 patients with UWS) were excluded from the present analysis. Patients were clinically investigated once a week during in-patient stay using standardized scales, i.e. the Coma Recovery Scale-Revised (CRS-R) [34] and the Wessex Head Injury Matrix [35]. A summary of all 25 included patients (8 patients in MCS; 17 patients with UWS) is displayed in Table 1. The mean age of the group of UWS patients (12 men; 5 women) was 52 years with a range from 29 to 78 years. The mean age of the group of MCS patients (7 men; one woman) was 48 years with a range from 19 to 77 years. All patients participating in this study showed preserved auditory functioning, largely preserved brainstem reflexes, and a fairly preserved sleep-wake-cycle based on assessments of the neurologists in charge. Further, none of the patients have been artificially ventilated nor sedated at time of scanning.

**Table 1:** Patients' information.

Subjects	Sex	Age	Etiology	Time since onset (in days)	At the time of fMRI	
					Diagnosis	CRS-R
*MCS*						
MCS01	M	77	Hypoxic	34	MCS	0/2/1/1/0/0
MCS02	M	59	Traumatic	85	MCS	3/3/1/1/0/3
MCS03	F	46	Traumatic	2960	MCS	1/2/2/1/0/1
MCS04	M	51	Traumatic	102	MCS	2/3/1/1/0/2
MCS05	M	37	Hypoxic	69	MCS	1/2/2/1/0/2
MCS06	M	47	Traumatic	49	MCS	1/2/1/1/1/2
MCS07	M	47	Traumatic	52	MCS	2/2/1/1/0/2
MCS08	M	61	Traumatic	116	MCS	3/0/4/1/0/2
*UWS*						
UWS01	M	59	Traumatic	116	UWS	1/0/1/1/0/0
UWS02	M	47	Hypoxic	27	UWS	0/0/1/0/0/0
UWS03	M	44	Traumatic	1456	UWS	0/0/1/1/0/0
UWS04	M	68	Hypoxic	74	UWS	1/0/1/1/0/0
UWS05	M	36	Traumatic	347	UWS	0/0/1/0/0/0
UWS06	M	50	Hypoxic	204	UWS	1/0/2/1/0/2
UWS07	M	69	Hypoxic	58	UWS	1/0/2/1/0/2
UWS08	F	39	Hypoxic	74	UWS	1/1/1/0/0/1
UWS09	M	47	Hypoxic	65	UWS	2/1/2/1/0/2
UWS10	F	29	Traumatic	105	UWS	1/0/2/1/0/0
UWS11	M	78	Hypoxic	39	UWS	1/0/0/1/0/0
UWS12	F	47	Traumatic	51	UWS	1/0/2/1/0/0
UWS13	M	63	Hypoxic	16	UWS	1/0/1/1/0/0
UWS14	M	51	Hypoxic	30	UWS	0/0/0/1/0/0
UWS15	M	50	Traumatic	165	UWS	1/0/2/1/0/1
UWS16	F	51	Hypoxic	1470	UWS	1/0/2/1/0/2
UWS17	F	49	Hypoxic	40	UWS	1/0/2/1/0/2

### Stimuli

Control subjects and patients were scanned while listening to 64 short sentences in 2 sessions containing a true or false meaning (e.g. ‘strawberries are red’ vs. ‘strawberries are blue’) created to attract attention. Sentences were recorded in German language by an Austrian male speaker with Cool Edit Pro 2.00 (1992–2000 Syntrillium Software Corporation). Stimuli were presented via headphones with Presentation software (Neurobehavioral Systems) in a pseudo-randomized order in 2 sessions (block-design: 8 blocks in each session; duration of each block: 16 sec; 4 sentences in each block). Control subjects and patients were instructed to lie still and carefully listen to the sentences.

### Data acquisition

Because data acquisition of patients was performed over a long period of time, patients' and controls' fMRI data were acquired using a 1.5 T scanner (Philips Gyroscan Intera) and two 3 T scanners (Philips Achieva and Siemens TIM TRIO) due to hardware changes at the clinical setting. Number of control subjects and patients were matched for field strength. Six control subjects, 2 patients in MCS and 4 with UWS were scanned with the 1.5 T Philips scanner. Four control subjects, 4 MCS and 10 UWS patients were scanned with the 3 T Philips scanner. For both Philips scanners, 134 T2*-weighted images were obtained with a gradient echo-planar sequence (EPI) in axial plane (25 slices with a thickness of 4.5 mm and an inter-slice gap of 0.5 mm; matrix size = 64×64; FoV = 210 mm^2^; TR = 2200 ms; TE = 45 ms; flip angle = 90°). The data of the remaining 15 control subjects, 2 MCS and 3 UWS patients were acquired with the 3 T Siemens scanner. Again, 134 T2*-weighted images were obtained with a gradient EPI in axial plane (25 slices with a thickness of 4.5 mm and an inter-slice gap of 0.5 mm; matrix size = 80×80; FoV = 210 mm^2^; TR = 2200 ms; TE = 30 ms; flip angle = 70°). In addition, T1-weighted MPRAGE sequences for anatomic information were acquired for each participant.

### Data analysis

Functional data were preprocessed and analyzed using Statistical Parametric Mapping (version SPM5; Wellcome Department of Cognitive Neurology, London, UK; http://www.fil.ion.ucl.ac.uk/spm/). The first 6 functional scans were considered as dummy scans and were discarded. Preprocessing steps included the following procedures: realignment to compensate for motion; unwarping (adjustment for movement-related artifacts); normalization of an average image of 128 images onto the MNI EPI template (because of the partially severe lesions in the patients' brain, affine only normalization was performed, i.e., no nonlinear functions); spatial smoothing using a Gaussian Kernel of 5 mm full width at half maximum. Voxel extent was resized to 3×3×3 mm. For single-subject statistical analysis, voxel-wise statistical parametric maps were generated for each subject. Each stimulus onset was modeled by a canonical hemodynamic response function. Data were filtered with a high-pass cut-off of 128 sec. For minimization of the impact of artifacts in fMRI time series data, a general linear model (GLM) regression using weighted least squares in combination with an autoregressive approach was implemented during Restricted Maximum Likelihood (ReML) parameter estimation [36]. Since there were no differences between true and false sentences in regions of the DMN which showed significant deactivation in the control group at a threshold level of *p* = 0.01, uncorrected, sentences were pooled for statistical analysis. In 16 patients, one of the 2 sessions had to be excluded due to artifacts. In these cases, only the remaining session was used for further analysis. For group analysis, subject-specific contrast images were entered into a voxel-based 2^nd^ level analysis with scanner type as a covariate. All results for the group analysis were thresholded at p>0.05, corrected for FWE at a whole brain level. For activity analysis on the single-subject level, pooled sentences were contrasted against rest. To focus on deactivations within the DMN, data were masked with the original image of the meta-analysis of DMN functional heterogeneity supplied by Angela R. Laird [23]. In addition to the masked voxel-based analysis, a region of interest (ROI) analysis was accomplished by extracting the mean contrast estimates at whole brain level for each main region of the default mode network and each participant (with a sphere of 6 mm radius) to perform *t*-tests and univariate analysis of variance. To account for differences between control subjects and patients, scanner type was implemented as a covariate. Additionally, mean contrast estimates were extracted for the following 3 regions which are involved in speech processing [37]: left superior temporal gyrus, left inferior frontal gyrus (lIFG), and the left precentral gyrus. Regions of interest within the DMN were chosen as follows: medial parietal (precuneus and PCC); medial frontal (vACC and medial prefrontal cortex); right middle temporal gyrus (rMTG); left middle temporal gyrus (lMTG). For the exact coordinates see Table S1. This selection was considered because medial parietal and frontal, as well as middle temporal regions are regarded as the most essential for the DMN [24]. It should be noted that Fox and Raichle define these middle temporal regions as lateral parietal cortex in their review. However, the coordinates stated by Shulman et al. [28] are quite identical with those labeled as middle temporal gyrus by Laird et al. [23]. In addition to the level of deactivation, the extension of the deactivation pattern can also be a subject of interest and may provide further information. Therefore, the number of deactivated voxels in each region was also investigated. ROI analysis was performed with SPSS (version 14; SPSS inc.; www.spss.com). Spearman correlation between time of onset and contrast estimates of each region, two-tailed, and Pearson correlation between CRS-R scores of the patients and contrast estimates of each region, one-tailed, were performed as well. To see if there was a relation between an absence of deactivation pattern and no response to auditory stimuli, contrast estimates between activation in the left superior temporal gyrus and deactivation in the DMN in patients were correlated. Because gender has an influence on brain networks [38] and because there are proportional sex differences between the control group and the patients' groups, additional *t*-tests were calculated for differences in contrast estimates for each region of the DMN between men and women. The same was done for potential differences in etiology for the patients' groups.

## Results

### Voxel-based analysis at group level

**Figure 1:**
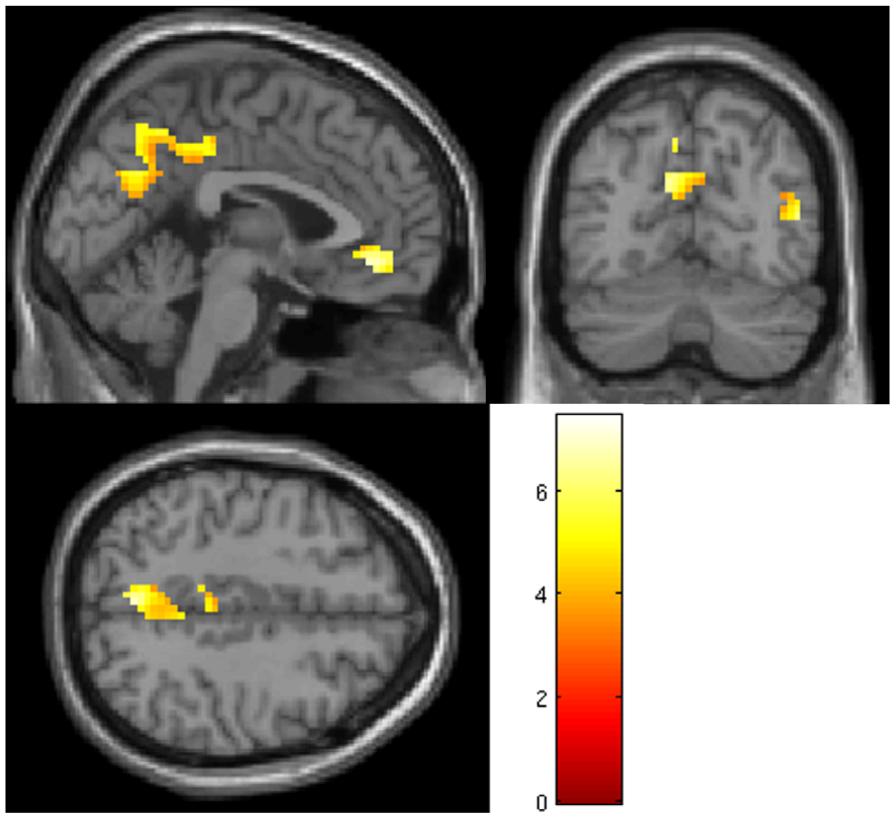
Deactivation of the DMN in the control group during sentence processing. Images display BOLD signal changes overlaid on a canonical template and transformed into standard MNI space. For display purposes, results are thresholded at *p*<0.001, uncorrected.

The control group showed wide-spread deactivation in medial parietal regions, *t*(23) = 7.43, *p* = 0.005, and medial frontal regions, *t*(23) = 7.16, *p*<0.009, all corrected for family-wise error (FWE). Deactivation in the rMTG and lMTG were not significant for multiple corrections. Results are displayed in Figure 1. The MCS and the UWS group, in contrast, failed to show a significant deactivation effect. The MCS group showed deactivation only when lowering the threshold to p = 0.01, uncorrected. The UWS group, in contrary, showed no deactivation at all.

### ROI analysis at group level

**Figure 2:**
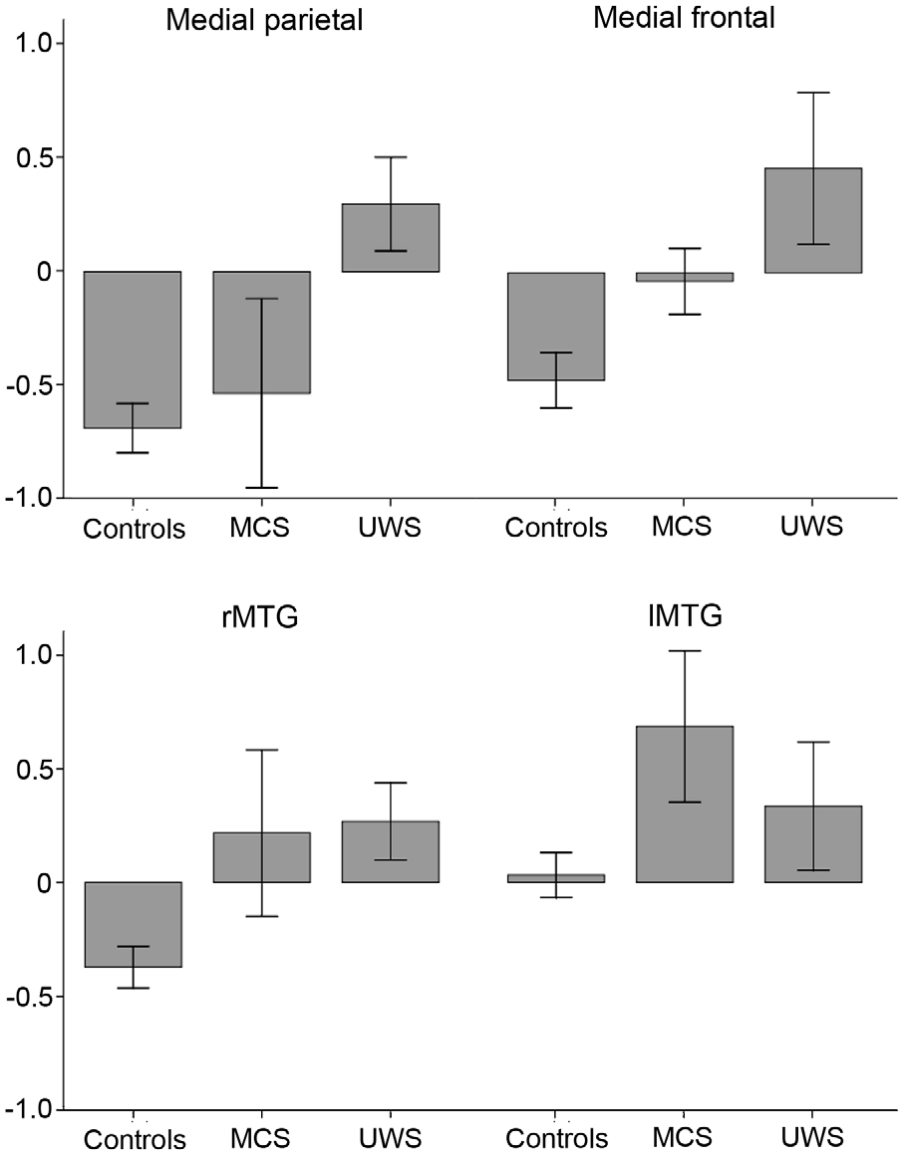
Strength of deactivation during sentence processing between groups. Image displays deactivation in healthy controls, patients in minimally conscious state (MCS), and patients with unresponsive wakefulness syndrome (UWS) in the 4 main regions of the DMN: medial parietal regions; medial frontal regions; right middle temporal gyrus (rMTG); left middle temporal gyrus (lMTG). Bars represent mean contrast estimates and standard error of mean.

Results of the ROI analysis are displayed in Table 2 and Figure 2. A main effect of group for contrast estimates was detected in medial parietal regions and in the rMTG. The control group showed significant deactivation in both medial regions and in the rMTG. The MCS and UWS group demonstrated no significant deactivation. Comparing contrast estimates between groups, control subjects demonstrated more deactivation in rMTG and lMTG compared to patients in MCS; and more deactivation in medial parietal regions, in medial frontal regions, and in the rMTG compared to patients with UWS. Between MCS and UWS patients, a trend towards differences in contrast estimates could be detected in medial parietal regions but was not significant when adjusting for multiple corrections. Results concerning the number of deactivated voxels did not offer any additional information (data not shown).

**Table 2:** Results from region of interest analysis.

	Contrast estimates		
Anatomical Regions	*F*-value	*t*-value	*p*-value*
*Controls*			
Medial parietal		−6.31	>0.001
Medial frontal		−3.89	0.004
rMTG		−4,05	>0.001
lMTG		0.74	1
*MCS*			
Medial parietal		−1.28	0.960
Medial frontal		−0.59	1
rMTG		−0.60	1
lMTG		2.06	0.312
*UWS*			
Medial parietal		1.45	0.672
Medial frontal		1.38	0.744
rMTG		1.59	0.528
lMTG		1.19	1
*All groups*			
Medial parietal	7.81		0.004
Medial frontal	3.76		0.124
rMTG	5.27		0.036
lMTG	1.53		0.228
*Controls vs. MCS*			
Medial parietal		−0.51	0.612
Medial frontal		−1.89	0.068
rMTG		−2.28	0.032
lMTG		−2.59	0.016
*Controls vs. UWS*			
Medial parietal		−4.58	<0.001
Medial frontal		−2.99	0.020
rMTG		−3.59	0.004
lMTG		−1.16	0.968
*MCS vs. UWS*			
Medial parietal		−2.02	0.055
Medial frontal		−1.21	0.239
rMTG		−0.15	0.886
lMTG		0.74	0.465

MCS, patients in minimally conscious state; UWS, patients with unresponsive wakefulness syndrome; rMTG, right middle temporal gyrus; lMTG, left middle temporal gyrus; DMN, default mode network;

*adjustment for multiple comparisons.

### Additional analysis at group level

A correlation between CRS-R scores of the patients and contrast estimates in medial parietal regions (r = 0.380, p = 0.031) and in medial frontal regions (r = 0.61, p = 0.001) could be detected. Correlations of the rMTG and lMTG were not significant. Differences in contrast estimates between patients with traumatic origin and patients with hypoxic origin were not significant, *t*(23)<1.66, *p*>0.11. Furthermore, correlations between time since onset and contrast estimates were not significant, *r*<0.21, *p*>0.31. Sex differences in contrast estimates were not significant, *t*(58)<1.931, *p*>0.24, either.

### Voxel-based analysis at the single-subject level

Results of the voxel-based analysis at the single-subject level revealed that all controls deactivated in the DMN during sentence processing at a threshold level of *p*<0.001, uncorrected (see Table S2). The patients, in contrast, exposed an abnormal and reduced deactivation pattern: Only 8 patients (32%), i.e., 2 patients in MCS (25%) and 6 patients with UWS (35%), showed signs of deactivation. Patients with a deactivation pattern showed a significantly reduced number of deactivated voxels in the DMN as a whole compared to controls, *t*(31) = 3.05, *p*<0.005. All subjects of the control group and all patients except for 3 with UWS showed activation in the left superior temporal gyrus during stimuli presentation at a threshold level of *p*<0.001, uncorrected (see Figure S1 to compare activation between patients). Correlations of the contrast estimates between activation in the left superior temporal gyrus and deactivation in the DMN in patients were not significant (*r*(25) = 0.183, *p* = 0.381). Interestingly, all patients showing a deactivation pattern demonstrated further activation in left frontal regions (*p*<.001, uncorrected), i.e. in the left inferior frontal gyrus and the left precentral gyrus, except for one patient with UWS (UWS16) who had large lesions in exactly these regions (see Figure S2). Additionally, 6 out of 17 patients who did not show a deactivation pattern (3 in MCS; 3 with UWS) also demonstrated activation in these areas (see Table S2). To confirm that activation in left frontal regions and deactivation in the DMN are related in patients, an additional Yates' chi-square goodness of fit test was calculated, *χ^2^*(1) = 4.03, *p* = 0.045.

## Discussion

The results of our study indicate that deactivation in medial regions of the DMN is reduced in MCS and absent in UWS compared with healthy control subjects. At the single-subject level, 17 out of 25 patients (6 MCS patients; 11 UWS patients) did not show a significant deactivation pattern. However, all patients showing a deactivation pattern (except for one) demonstrated activation in left frontal regions during speech exposure. Further, a significant relation between the CRS-R scores of the patients and deactivation in medial parietal and medial frontal regions was identified.

First of all, it is important to state that most of the patients did not express a significant deactivation pattern while almost all of them demonstrated activation in the superior temporal gyrus during speech exposure (except for 3 UWS patients, see Figure S1). Thus, an absence of deactivation cannot be explained by the means of a broad lack of reactivity to auditory presented speech. Consistently, correlations between activation in the left superior temporal gyrus and deactivation in the DMN were not significant. Subjects in deep sedation, for example, also show activation in temporal regions during language processing but no activation in prefrontal regions, which are associated with higher-level semantic processing [37]. The authors relate the reduced activity in prefrontal areas to reduced awareness of speech, and the preserved activity in temporal regions to unconscious processing. Consequently, regions of the DMN do not simply deactivate due to an unconscious processing of auditory presented sentences.

Second, although it is not understood what activity of the DMN during the resting state really implies, it seems reasonable that deactivation in medial regions is linked to a functional interruption of an ongoing mental stream – whatever it may indicate – to make resources available that are necessary to focus attention on the demands of the task [39], [40]; and that this interruption goes along with a more conscious processing of stimuli allowing the person to remain aware of external events [41]. In line with these considerations, the process of interruption appears to be reduced in MCS patients compared to healthy controls and absent in patients with UWS as displayed in Figure 2. The significant relationship between CRS-R scores of the patients and the level of deactivation within medial parietal and medial frontal regions confirms the assumption that the ability to deactivate is associated with the level of consciousness. This may not be a surprising result because it corresponds with findings of previous studies investigating functional DMN connectivity in patients with DOC [30], [42]. However, the interesting question here is does deactivation provide additional information in respect to differentiation of diagnosis in DOC patients?

While investigation of DMN connectivity demonstrates a relation to the level of consciousness, it can still be identified in altered states of consciousness like in deeply anaesthetized monkeys [43], and in healthy subjects during light sedation [44] and during sleep [45]. These findings imply that resting state connectivity cannot exclusively reflect the level of consciousness (see Boly et al. [33] for a review). In contrast, deactivation of the DMN is a response to external stimuli which most likely occurs in the course of target-directed and probably attention-focused processing. Thus, the ability to deactivate is based on more specific processes of perception. In our study investigating deactivation of the DMN, a few patients with the ability to deactivate could be detected and distinguished from others who did not demonstrate deactivation. All control subjects but only 2 patients in MCS and 6 with UWS showed a deactivation pattern. Both MCS patients showed a wide-spread deactivation pattern quite similar to those of controls, which was present even when correcting for FWE at a threshold level of p = 0.05 (Figure S3). Besides, all patients with a deactivation pattern exposed activation in areas involved in higher-order language processing during auditory sentence presentation (except for one who had large lesions in the lIFG and left precentral gyrus as shown in Figure S2). Correspondingly, results of the chi-square test indicate an association between deactivation in the DMN and activation in left frontal regions in patients. Activation in these areas is considered to reflect conscious awareness of speech [37]. This suggests that patients who deactivate may have some preserved functions of conscious processing.

On the other hand, 6 other patients also showed activation in these prefrontal and precentral regions during speech processing although they did not demonstrate task-induced deactivation. But this finding actually corresponds quite well with the hypothesis that deactivation of the DMN is associated with an interruption of ongoing processing to focus attention. If attention is focused during a language task, higher-order language processing will be the consequence. On the contrary, attention focusing is probably not a necessary requirement for higher-order language processing. This could be an explanation for the finding that principally all patients with a deactivation pattern showed fairly preserved activation in frontal areas, while not all patients with preserved frontal activity in response to speech showed deactivation within the DMN.

A limitation to this study is that the difference between MCS and UWS patients in medial regions is not significant when adjusting for multiple corrections. Hence, the difference between MCS and UWS can only be stated as a trend. In conjunction with the association between CRS-R scores and deactivation in the patient group, though, we conclude that there is indeed a relation between deactivation in the DMN and the level of consciousness. Patients are divided into groups based on cut-off values. In reality though, UWS and MCS are a continuum and not as clearly dissociable as it may seem.

However, the present interpretation seems to stand in contrast to the results at single-subject level. At group level, there is a correspondence between diagnosis and CRS-R scores, respectively, and deactivation in the DMN. When looking at the single-subject level, though, the percentage of patients showing a deactivation pattern is a bit higher in the UWS group (25% of MCS patients and 35% of UWS patients). When considering that those 2 analyses are based on 2 different dimensions, this inconsistency becomes more understandable. At the single-subject level, the existence of a condition, in particular a deactivation pattern is measured exceeding a given cut-off. At the group level, the strength of deactivation as a continuum is reflected independent of its significance. Besides, this contradicting distribution between MCS and UWS corresponds empirically very well with previous studies investigating active and passive paradigms at a single-subject level. In the study by Monti et al. [8], 17% of the UWS patients but only 3% of the MCS patients were able to willfully modulate their brain activity. Coleman and co-workers [46] found similar neuronal markers for language processing in about 40% of the patients in both patients' groups. The authors argue that although fMRI responses are only neuronal correlates and do not show a causal relationship between response and performance, they can be a reliable indicator for preserved functions independent of the distinction between MCS and UWS.

An additional question is concerning the specificity of deactivation within the DMN. We have to keep in mind that deactivation is altered in various mental diseases and pathological states like Alzheimer's disease [47], autism [48], and schizophrenia [49]. However, this is not a specific problem of impaired deactivation but applies to alterations in the DMN in general (for further review see Broyd et al. [50]). Future studies must explore if there are specific variations in properties of the DMN in patients with DOC compared to other disorders like, e.g., Alzheimer's disease.

In conclusion, the 3 main features of the default mode network in patients with DOC are impaired: metabolism; functionality; task-induced deactivation. MCS patients showed reduced deactivation, while patients with UWS did not obtain any deactivation pattern at all. Task-induced deactivation in medial regions of the DMN seems to correspond with the level of consciousness. On the single-subject level, patients with a deactivation pattern demonstrated preserved activation in lIFG or left precentral regions, which are associated with conscious processing [37]. Hence, in addition to analyzing connectivity of the resting state network, which correlates with the level of consciousness [30], and metabolism, which is reduced [16], [29], investigating task-induced deactivation gives the opportunity to differentiate by selecting those DOC patients with the ability to interrupt ongoing mental processes to focus attention. Thus, the presence of a deactivation pattern may supply additional evidence for conscious processing and, therefore, may be quite suitable as a marker of preserved aspects of consciousness. In future studies, deactivation should be investigated in relation to the other features of the DMN to receive a more detailed picture of resting state networks and their function in DOC.

## Supporting Information

Figure S1 **Activation during sentence processing in patients in the left superior temporal gyrus.** Images display BOLD signal changes overlaid on the structural template of each patient and transformed into standard MNI space. Results are thresholded at *p*<0.001, uncorrected.
(TIF)

Figure S2 **Activation in left inferior frontal and left precentral gyrus in patients with a deactivation pattern.** Images display BOLD signal changes overlaid on the structural template of each patient and transformed into standard MNI space. Results are thresholded at *p*<0.001, uncorrected. Circles show wide-spread lesions in frontal regions of patient UWS16.
(TIF)

Figure S3 **Deactivation pattern of MCS07 and MCS08.** Images display BOLD signal changes overlaid on the structural template of each patient and transformed into standard MNI space. Results are thresholded at *p*<0.001, uncorrected.
(TIF)

Table S1 **Coordinates of region of interest.**
(PDF)

Table S2 **Sum of deactivated voxels within the DMN and sum of activated voxels within areas of higher-order speech processing for controls and patients.**
(PDF)
