# Supplementary figures and images for: Deactivation of the Default Mode Network as a Marker of Impaired Consciousness: An fMRI Study
Source: PLoS One. 2011 Oct 19;6(10):e26373. doi: 10.1371/journal.pone.0026373 (PMC3198462; doi:10.1371/journal.pone.0026373)

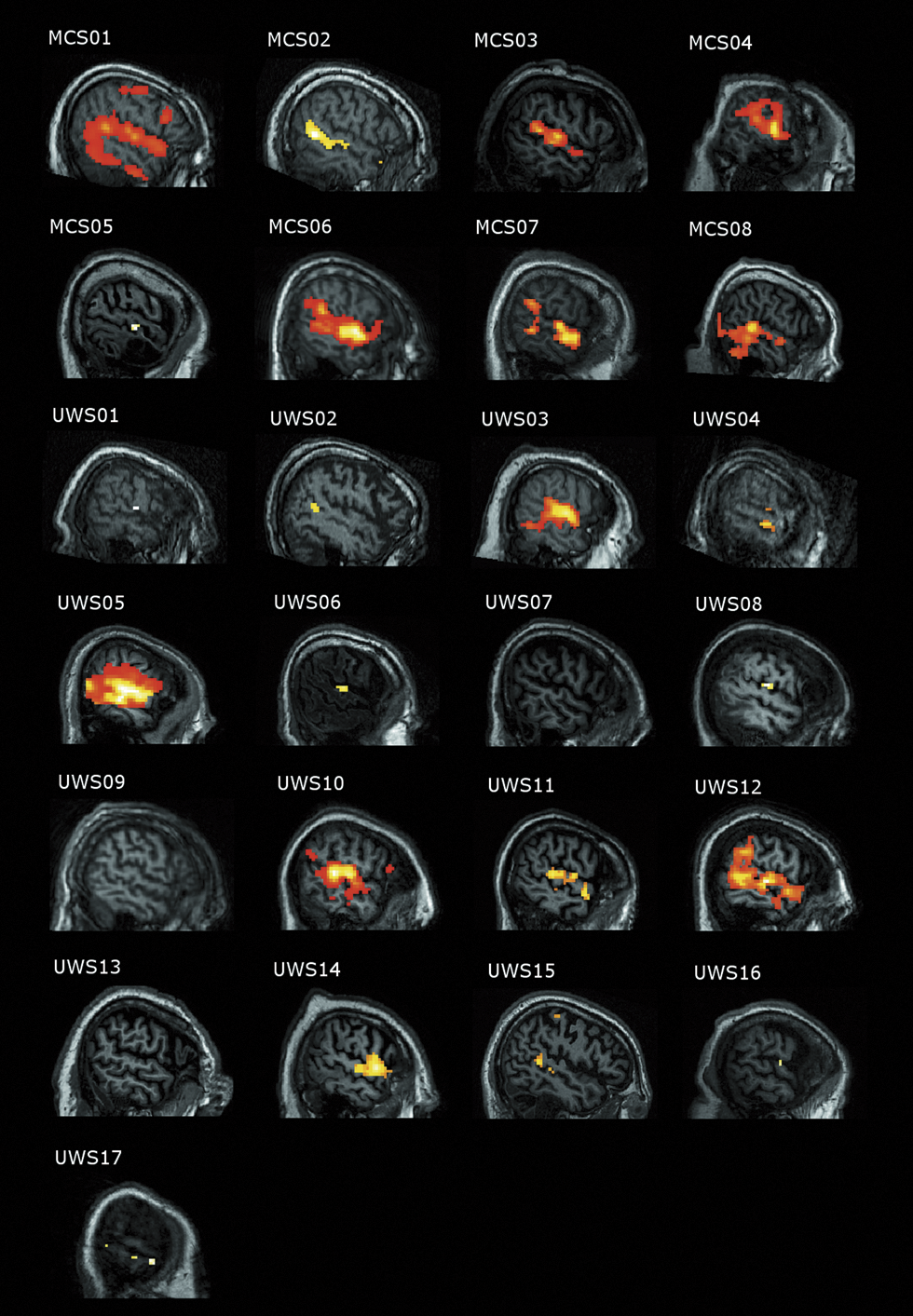

Supplement: Figure S1 — Activation during sentence processing in patients in the left superior temporal gyrus. Images display BOLD signal changes overlaid on the structural template of each patient and transformed into standard MNI space. Results are thresholded at p<0.001, uncorrected. (TIF) [file pone.0026373.s001.tif]

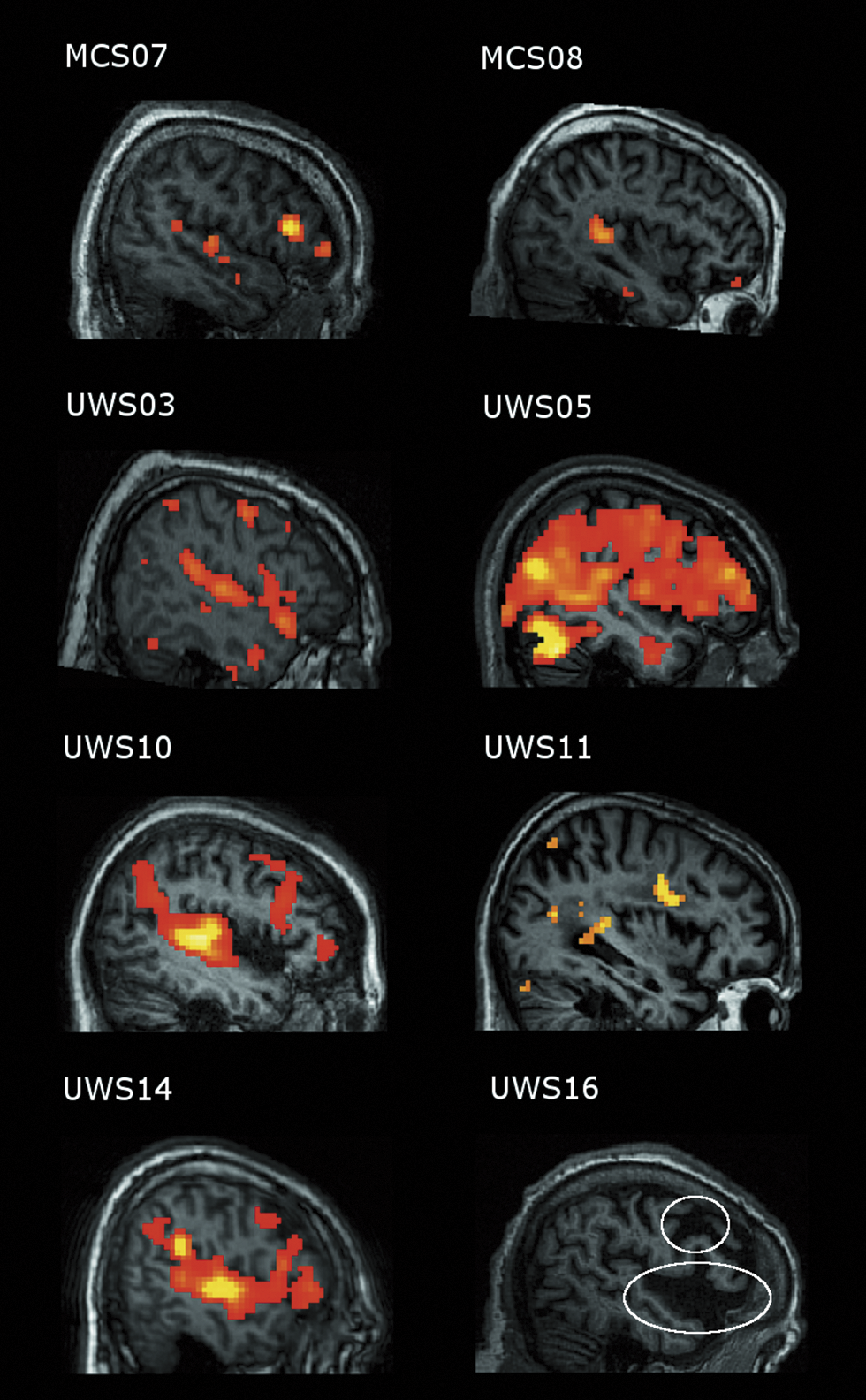

Supplement: Figure S2 — Activation in left inferior frontal and left precentral gyrus in patients with a deactivation pattern. Images display BOLD signal changes overlaid on the structural template of each patient and transformed into standard MNI space. Results are thresholded at p<0.001, uncorrected. Circles show wide-spread lesions in frontal regions of patient UWS16. (TIF) [file pone.0026373.s002.tif]

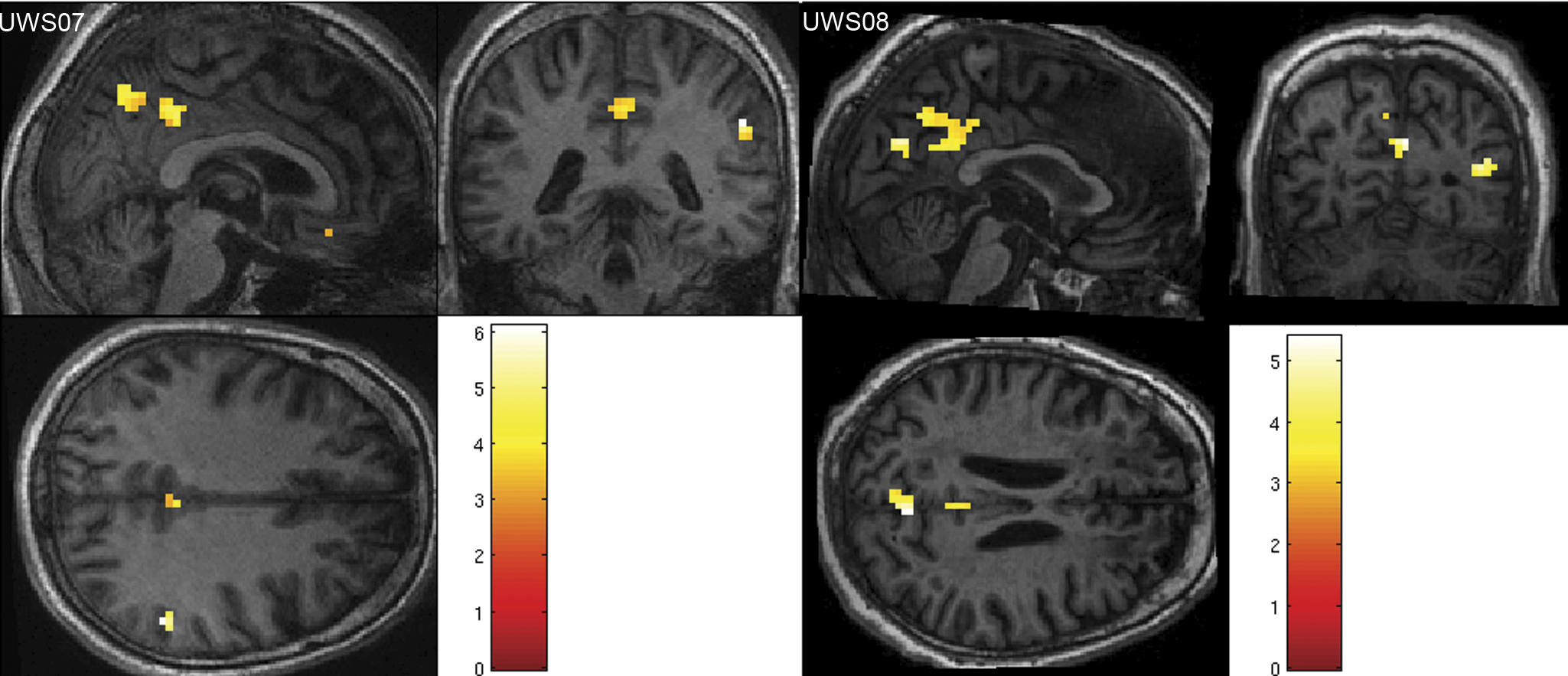

Supplement: Figure S3 — Deactivation pattern of MCS07 and MCS08. Images display BOLD signal changes overlaid on the structural template of each patient and transformed into standard MNI space. Results are thresholded at p<0.001, uncorrected. (TIF) [file pone.0026373.s003.tif]
