# Supplementary material for: Deactivation of the Default Mode Network as a Marker of Impaired Consciousness: An fMRI Study
Source: PLoS One. 2011 Oct 19;6(10):e26373. doi: 10.1371/journal.pone.0026373 (PMC3198462; doi:10.1371/journal.pone.0026373)
Supplement: Table S1 — Coordinates of region of interest. (PDF) [file pone.0026373.s004.pdf]

Table S1. Coordinates of region of interest

Anatomical Regions		MNI Coordinates (x/y/z)
Medial parietal	*Precuneus*	0/-56/50
	Posterior cingulate cortex	0/-52/25
Medial frontal	*Ventral anterior cingulate cortex*	0/36/0
	Medial prefrontal cortex	0/57/10
Right middle temporal gyrus		51/-67/19
Left middle temporal gyrus		-44/-67/-19
Left superior temporal gyrus		-60/-30/0
Left inferior frontal gyrus		-44/22/-12
Left precentral gyrus		-50/16/30
