# Supplementary material for: Deactivation of the Default Mode Network as a Marker of Impaired Consciousness: An fMRI Study
Source: PLoS One. 2011 Oct 19;6(10):e26373. doi: 10.1371/journal.pone.0026373 (PMC3198462; doi:10.1371/journal.pone.0026373)
Supplement: Table S2 — Sum of deactivated voxels within the DMN and sum of activated voxels within areas of higher-order speech processing for controls and patients. (PDF) [file pone.0026373.s005.pdf]

Table S2. Sum of deactivated voxels within in the DMN and sum of activated voxels within areas of higher-order speech processing for controls and patients

	**Deactivation**	**Activation**	
	**DMN**	**lIFG**	**Left precentral**
Controls			
a01	5	1492	129
a02	93	1664	6
a03	96	1332	121
a04	28	1597	46
a05	36	14	102
a06	56	3379	1066
a07	290	11439	v.s.
a08	130	3445	v.s.
a09	205	4609	1244
a10	3	6	32
a11	356	1017	v.s.
a12	1	5475	v.s.
a13	44	2107	5923
a14	214	4918	v.s.
a15	81	14	450
a16	444	3144	v.s.
a17	1	3994	v.s.

a18	479	3024	v.s.
a19	18	6534	v.s.
a20	79	5647	v.s.
a21	313	48	2689
a22	61	5560	1
a23	49	2844	v.s.
a24	147	3064	v.s.
a25	239	1381	617
MCS			
MCS01	0	0	75
MCS02	0	0	0
MCS03	0	0	0
MCS04	0	19	23
MCS05	0	0	0
MCS06	0	1612	149
MCS07	85	0	71
MCS08	122	4	0
UWS			
UWS01	0	0	0
UWS02	0	0	0
UWS03	3	931	18

UWS04	0	6	25
UWS05	3	25864	v.s.
UWS06	0	0	0
UWS07	0	0	0
UWS08	0	0	24
UWS09	0	0	0
UWS10	23	0	285
UWS11	20	0	2536
UWS12	0	3012	v.s.
UWS13	0	0	0
UWS14	1	490	3
UWS15	0	0	0
UWS16	60	0	0
UWS17	0	0	0

MCS, patients in minimally conscious state; UWS, patients with unresponsive wakefulness syndrome; DMN, default mode network; lIFG, left inferior frontal gyrus; v.s., see above (same cluster as lIFG)
